# Appendiceal neurofibroma after resection of multiple gastrointestinal stromal tumors of the small intestine in a patient with neurofibromatosis type 1: a case report

**DOI:** 10.1186/s40792-024-02062-x

**Published:** 2024-11-15

**Authors:** Katsuya Sakashita, Shoichi Manabe, Akio Shiomi, Hiroyasu Kagawa, Yusuke Yamaoka, Shunsuke Kasai, Yusuke Tanaka, Takuma Oishi, Teiichi Sugiura

**Affiliations:** 1 Division of Colon and Rectal Surgery, Shizuoka Cancer Center, 1007 Shimonagakubo, Nagaizumi-Cho, Sunto-Gun, Shizuoka, 411-8777 Japan; 2 Division of Hepato-Biliary-Pancreatic Surgery, Shizuoka Cancer Center, Shizuoka, Japan; 3 Division of Diagnostic Pathology, Shizuoka Cancer Center, Shizuoka, Japan

**Keywords:** Neurofibromatosis type 1, Appendiceal neurofibroma, Gastrointestinal stromal tumor

## Abstract

**Background:**

Neurofibromatosis type 1 (NF1), also known as von Recklinghausen disease, is an autosomal dominant disorder that can affect multiple organs. Although gastrointestinal manifestations, such as neurofibromas and gastrointestinal stromal tumors (GISTs), can occur, appendiceal neurofibromas are extremely rare, with no documented cases of their occurrence following other gastrointestinal lesions. Herein, we report a case of an appendiceal neurofibroma following the resection of multiple small intestinal GISTs.

**Case presentation:**

A 68-year-old man with NF1 presented with melena and was diagnosed with anemia due to bleeding from multiple small intestinal GISTs. Laparoscopic three partial resection of the small intestine was performed to control the bleeding. Histopathologic examination revealed the proliferation of spindle cells that are positive for c-kit and Discovered on GIST-1, confirming the diagnosis of GIST. Two years later, a follow-up computed tomography (CT) scan revealed a progressively enlarging mass in the appendix with suspected invasion into the small intestine. Positron emission tomography/CT showed fludeoxyglucose accumulation in the tumor. Therefore, considering the possibility of malignancy, laparoscopic ileocecal resection with lymph node dissection was performed. Postoperatively, melena was observed, but the anemia did not progress and improved with fasting and hemostatic therapy. The patient was eventually discharged on postoperative day 8. Histopathologic examination revealed spindle cell proliferation with positivity for S-100, confirming the diagnosis of neurofibroma.

**Conclusions:**

Patients with NF1 can develop a variety of gastrointestinal lesions. Appendiceal neurofibroma can be difficult to diagnose preoperatively and differentiate from malignancy. Hence, surgical resection should be considered.

## Background

Neurofibromatosis type 1 (NF1), also known as von Recklinghausen disease, is an autosomal dominant neurocutaneous syndrome. NF1 is a multisystem disorder that can affect any organ in the body. The most typical clinical manifestations include neurofibromas and café au lait spots [1]. Gastrointestinal lesions are reported in 10–25% of patients with NF1 [2]. The most common sites for gastrointestinal lesions are the small intestine (43.6%) and stomach (41.0%) [3]. However, neurofibromas of the appendix are extremely rare, with only a few cases documented in the literature. Furthermore, to the best of our knowledge, no study has reported their coexistence with other gastrointestinal lesions.

Herein, we report a case of a patient with NF1 who developed an appendiceal neurofibroma following the resection of multiple gastrointestinal stromal tumors (GISTs) in the small intestine.

## Case presentation

A 68-year-old man diagnosed with NF1 presented to the previous hospital with a chief complaint of melena. Laboratory tests revealed anemia, prompting admission for blood transfusion therapy. Upper and lower gastrointestinal endoscopy failed to identify any causative lesions, leading to his referral to our hospital for further evaluation and management.

**Fig. 1:**
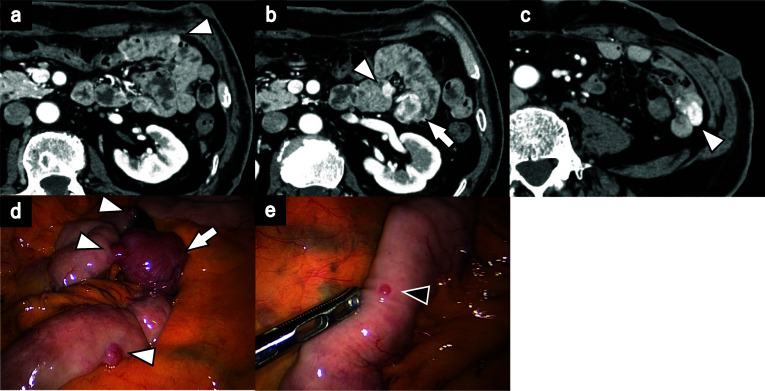
**a**–**c** Contrast-enhanced computed tomography findings. Axial image. Multiple contrast-enhancing masses were seen in the small intestine (white arrowhead). The largest mass was approximately 20 mm in diameter (white arrow). **d**, **e** Intraoperative findings. Multiple masses were seen in the small intestine (white arrowhead), with the largest mass located in the upper jejunum (white arrow). Multiple small lesions less than 5 mm in size were also seen (black arrowhead)

Upon arrival at our hospital, a physical examination revealed multiple cutaneous neurofibromas, but no abdominal symptoms were found. Blood tests confirmed anemia with a hemoglobin level of 8.7 g/dL. Due to the rapid progression of anemia caused by melena, blood transfusion was required. A computed tomography (CT) scan revealed multiple tumors in the small intestine, suggestive of small intestinal GISTs (Fig. 1). Capsule endoscopy identified bleeding in the jejunum and ileum. Double balloon upper gastrointestinal endoscopy revealed an ulcer on the surface of the jejunal tumor, which was thought to be the source of bleeding, so tattooing was performed. A double balloon endoscopy of the lower gastrointestinal tract was not performed. The lesion in the ileum could not be identified. Consequently, the patient was diagnosed with gastrointestinal bleeding due to multiple small intestinal GISTs based on clinical findings.

**Fig. 2:**
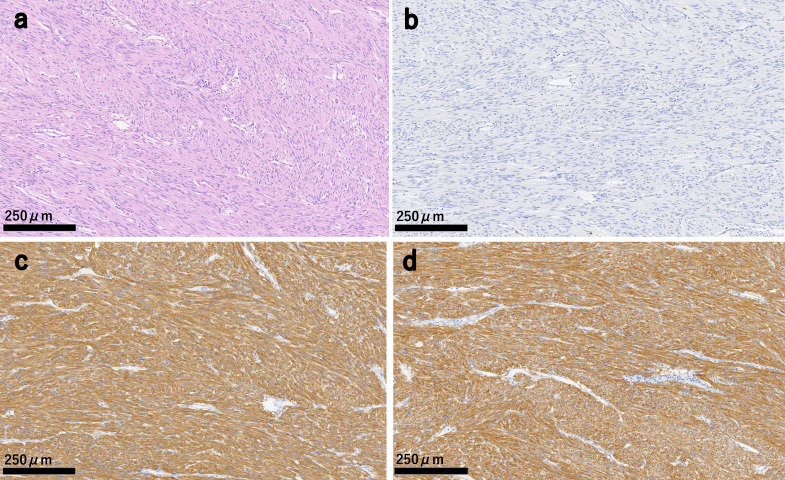
Microscopic findings of the resected specimen. **a** Proliferation of spindle-shaped cells is noted within the small intestinal wall. Hematoxylin and eosin staining. **b** Immunohistochemical staining for S-100 yielded negative results.** c** Immunohistochemical staining for the c-kit yielded a positive result. **d** Immunohistochemical staining for the DOG-1 yielded a positive result

Surgical intervention was deemed necessary to control the bleeding. Laparoscopic partial resection of the small intestine was performed at three sites. Initially, a 3 cm marked tumor in the upper jejunum was identified and resected. Approximately 50 cm distal to this site, a 10 mm tumor was identified and resected. In addition, a 5 mm tumor was identified 150 cm proximal to the end of the ileum, which was also resected as a possible source of bleeding. However, owing to the extensive number of small lesions less than 5 mm, complete excision was impractical, resulting in the retention of some small lesions in situ. The patient was discharged on the seventh postoperative day without developing any complications. Histopathological examination revealed tumors up to 30 mm in diameter within the small bowel wall, each demonstrating spindle cell proliferation. Immunohistochemical staining showed positivity for c-kit, Discovered on GIST-1 (DOG1), cluster of differentiation 34 (CD34), and desmin, while S-100 was negative. The Ki-67 labeling index was 5% (Fig. 2). Therefore, the patient was diagnosed with small intestinal GIST.

**Fig. 3:**
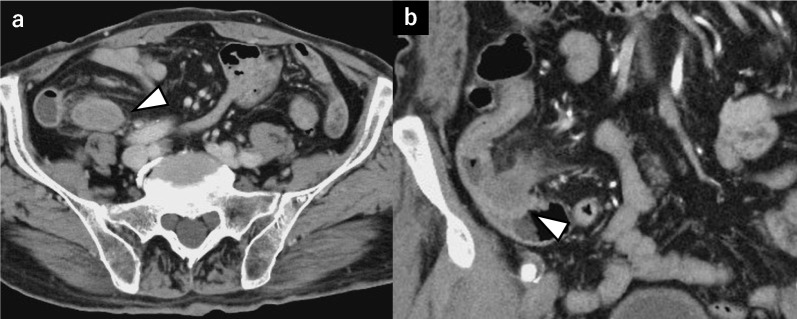
Contrast-enhanced computed tomography findings. **a** Axial image. A 27-mm mass is detected in the appendix. **b** Coronal image. The tip of the appendix is in contact with the small intestine, raising the possibility of tumor invasion

**Fig. 4:**
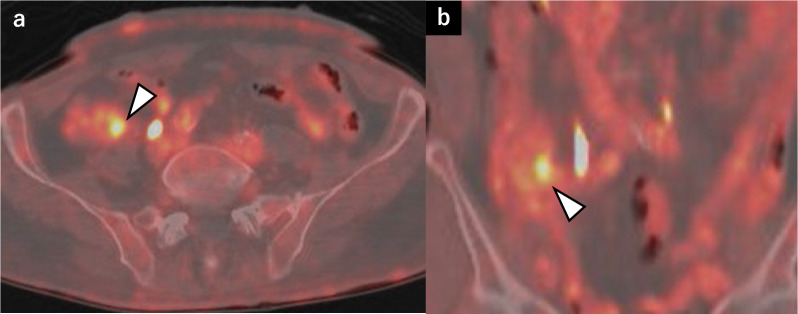
A positron emission tomography/computed tomography scan showed fluorodeoxyglucose accumulation with a maximum standardized uptake value of 5.05, which was consistent with the appendiceal mass

Two years later, a follow-up CT scan revealed a gradually expanding 27-mm tumor on the appendix, adjacent to the small intestine (Fig. 3). No symptoms of abdominal pain, vomiting, or diarrhea were observed. Laboratory tests yielded normal levels of carcinoembryonic antigen (1.7 ng/mL) and carbohydrate antigen 19–9 (10 U/mL). No elevated inflammatory response was observed. Lower gastrointestinal endoscopy showed mild redness and elevation at the appendiceal orifice but without a visible tumor. A positron emission tomography/CT (PET/CT) scan showed FDG accumulation with a maximum standardized uptake value of 5.05 corresponding to the appendiceal mass (Fig. 4). Surgical treatment was chosen due to the observed tumor growth, FDG uptake on PET/CT, and the suspicion of invasion into the small intestine. The preoperative diagnosis was appendiceal GIST, and surgery was planned. The differential diagnoses included appendiceal cancer and neurogenic tumor.

**Fig. 5:**
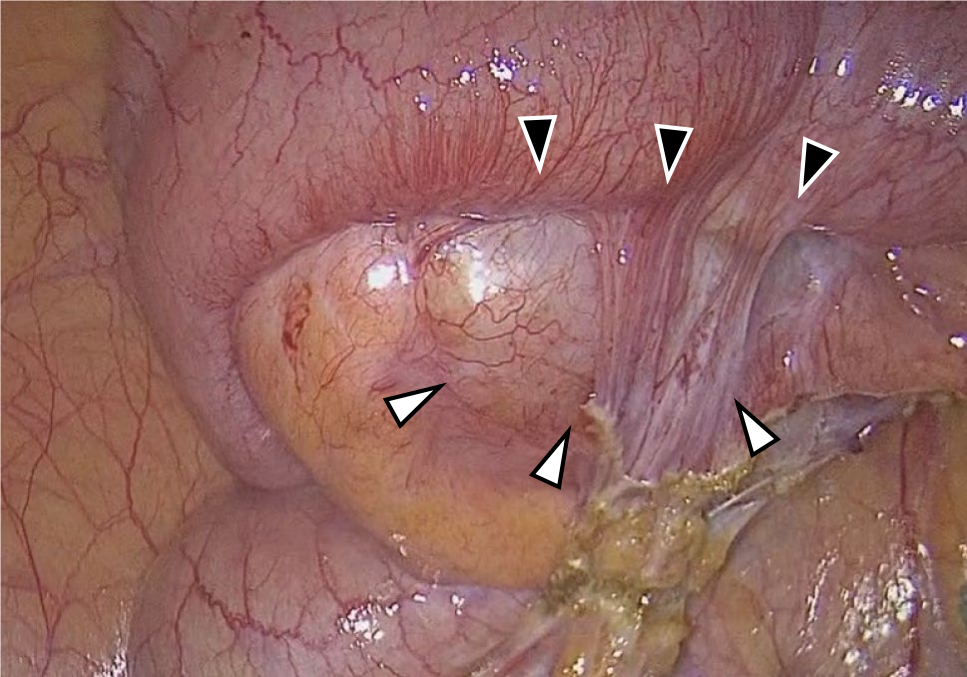
Intraoperative findings. A mass was observed in the appendix (white arrowhead). It was firmly adherent to the small intestine (black arrowhead), and infiltration was suspected

Intraoperative findings revealed an appendiceal mass lesion approximately 30 mm in size, with possible infiltration into the small intestine (Fig. 5). Considering the possibility of appendiceal cancer, laparoscopic ileocecal resection with lymph node dissection was performed. In addition, no significant change was noted in the small intestinal GIST on intraoperative observation. The patient experienced melena on postoperative day 2, recovered after fasting and hemostatic treatment, and was eventually discharged on postoperative day 8.

**Fig. 6:**
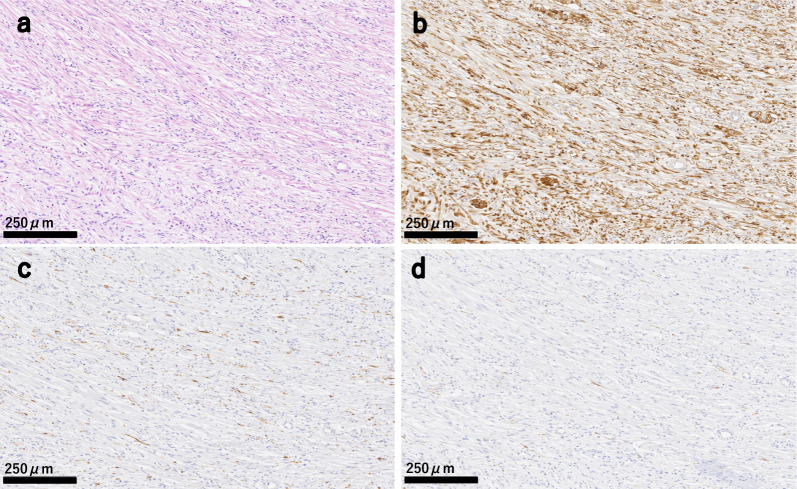
Microscopic findings of the resected specimen. **a** Spindle cell proliferation was observed in the appendix tumor. Hematoxylin and eosin staining. **b** Immunohistochemical staining for S-100 yielded a positive result. **c** Immunohistochemical staining for c-kit yielded a negative result. **d** Immunohistochemical staining for DOG-1 yielded a negative result

Histopathological analysis of the appendix detected a 42 × 24 mm tumor and spindle-shaped cell proliferation. No malignant findings were observed in the tumor and lymph nodes. Immunohistochemical staining showed positivity for S-100, desmin, α-smooth muscle actin, and CD34, while c-kit and DOG1 were negative. The Ki-67 labeling index was 6% (Fig. 6). Therefore, the diagnosis was appendiceal neurofibroma.

## Discussion

NF1, also recognized as von Recklinghausen disease, is one of the most prevalent autosomal dominant single-gene neurocutaneous disorders [4]. Although patients with NF1 typically present with characteristic skin lesions, gastrointestinal involvement can also occur, with neurofibromas or GISTs being the predominant histologic types [5]. GISTs are the most common gastrointestinal lesions in NF1, with an estimated prevalence of 5%–30% [6]. Although GISTs typically originate in the stomach, GISTs related to NF1 arise in the small intestine in approximately 90% of patients and frequently manifest as multiple lesions [7]. Resectable GISTs with a diameter of 2 cm or more are eligible for surgical resection, which remains the sole curative treatment option. In this case, the presence of anemia requiring blood transfusion led to the decision for early surgical intervention, and lower double-balloon endoscopy was consequently not performed. However, achieving an accurate preoperative diagnosis is important to avoid unnecessary bowel resection.

Neurofibromas in the abdomen in patients with NF1 can occur from the esophagus to the rectum, as well as in the peritoneum and mesentery [8]. Lesions are predominantly found in the jejunum, stomach, ileum, duodenum, and colon. These tumors can lead to complications such as obstruction, gastrointestinal bleeding, or perforation. Neurofibromas in the appendix are extremely rare, with only ten patients documented to date [9–18]. Among these patients, seven presented with abdominal pain, while only two remained asymptomatic, similar to our study patient. The most common preoperative diagnosis was appendicitis in six patients. Notably, only one case report has documented a preoperative diagnosis of neurofibroma, indicating the difficulty in establishing an accurate preoperative diagnosis. Furthermore, no case report has documented the development of other tumors in the digestive tract, as observed in our patient.

The preoperative diagnosis of appendiceal tumors in patients with NF1 is often challenging. Neurofibroma, which can occur at any site, should be considered as a potential differential diagnosis. However, in previous reports, preoperative diagnosis was not accurately made in most cases, underscoring the difficulty of making a precise preoperative diagnosis. Moreover, distinguishing between benign and malignant lesions can be challenging. Although neurofibromas are benign tumors, patients with NF1 are at increased risk of various types of malignancies [19]. Patients with NF1 have an 8–12% risk of developing malignant peripheral nerve sheath tumors [20]. Therefore, the possibility of malignancy should always be considered when evaluating gastrointestinal lesions in patients with NF1. In our patient, FDG accumulation was observed on PET/CT. It is already known that GIST shows uptake on PET/CT, and the preoperative imaging findings in this case were also consistent with GIST [21]. In patients with NF1, there have been reports indicating that FDG uptake is observed to some extent in superficial lesions, and the degree of FDG uptake is useful for identifying malignant lesions [22]. However, there are no significant reports regarding gastrointestinal lesions in NF1 patients, and no mention of PET findings in case reports of appendiceal neurofibroma. On the other hand, previous reports have documented cases presenting with inflammation, making it difficult to completely rule out the possibility that FDG uptake in this case could be related to some form of inflammation occurring during the clinical course. Therefore, the interpretation of the results should be done with caution, and further case accumulation is necessary in the future (see Table 1).

**Table 1:** Previous case reports of appendiceal neurofibroma in a patient with von Recklinghausen’s disease

No.	Authors	Age	Gender	Symptom	Preoperative diagnosis	Surgical procedure	Size (cm)
1	Merck and Kindblom [12]	24	M	Pain	Appendicitis	Appendectomy	NA
2	Olsen [13]	24	M	Pain	NA	Appendectomy	7 × 3
3	Samuel et al. [16]	19	M	Pain	NA	Appendectomy	3 × 7 × 8
4	Rosenberg et al. [15]	33	F	No	Intraoperative incidental finding	Appendectomy	12
5	Agaimy et al. [9]	45	M	NA	NA	Autopsy	0.3
6	Guo et al. [10]	62	F	Pain	Tubular mass from fallopian tube	Right hemicolectomy	9 × 7
7	Ozaki et al. [14]	51	M	Pain	Appendicitis	Appendectomy	4 × 3 × 3
8	Wilson et al. [18]	24	M	Pain	Appendiceal neurofibroma	Ileocecectomy	7 × 3
9	Komo et al. [11]	62	F	No	Cured appendicitis	Cecectomy	2 × 7
10	Van de Steen et al. [17]	74	M	Pain	Chronic appendicitis	Appendectomy	NA
11	Present case	68	M	No	Gastrointestinal stromal tumor	Ileocecectomy	4 × 2

NA: not available

Surgical resection is the standard of care for appendiceal neurofibroma. This treatment is aimed at improving symptoms, preventing complications, and avoiding malignant transformation [5]. Komo et al. performed a cecectomy to ensure negative surgical margins for an intraoperatively diagnosed appendiceal mass [11]. They stated that if the postoperative pathologic diagnosis had been appendiceal cancer or low-grade appendiceal mucinous neoplasm with positive margins, a secondary ileocecal resection with or without lymph node dissection would have been necessary. In this case, considering the possibility of malignant infiltration, laparoscopic ileocecal resection with lymph node dissection was performed. However, no consensus exists regarding appropriate surgical procedures including the necessity of performing lymph node dissection.

Patients with NF1 may present with multiple comorbidities that require careful monitoring. Patients with NF1 are approximately 2.7 times more likely to develop malignancies than the general population, highlighting the necessity for meticulous follow-up [23]. In addition, lesions exhibiting growth or associated with pain may indicate malignancy and warrant frequent evaluation [24]. The appendiceal neurofibroma was discovered incidentally in an asymptomatic patient during postoperative follow-up for GIST. This finding suggests that even asymptomatic patients may be at risk for developing additional lesions, indicating that heightened surveillance could be beneficial.

## Conclusion

Patients with NF1 can develop various types of gastrointestinal lesions, which can be challenging to diagnose. This case report documented a patient who developed multiple small intestinal GISTs followed by an extremely rare presentation of an appendiceal neurofibroma. The preoperative diagnosis of appendiceal neurofibroma is challenging, and distinguishing it from malignancy can be difficult, warranting consideration of surgical resection.

## Data Availability

The datasets used in the current study are available from the corresponding author upon reasonable request.

## Abbreviations

NF1: Neurofibromatosis type 1
GIST: Gastrointestinal stromal tumor
CT: Computed tomography
PET/CT: Positron emission tomography/computed tomography
